# Changes in neutrophile-to-lymphocyte ratio as predictive and prognostic biomarker in metastatic prostate cancer treated with taxane-based chemotherapy

**DOI:** 10.1007/s12672-022-00603-0

**Published:** 2022-12-15

**Authors:** Manuel Neuberger, Christel Weiß, Nora Goly, Janina Skladny, Katja Nitschke, Frederik Wessels, Karl F. Kowalewski, Frank Waldbillig, Friedrich Hartung, Malin Nientiedt, Luisa Egen, Jonas Herrmann, Jonas Jarczyk, Margarete Teresa Walach, Maximilian C. Kriegmair, Niklas Westhoff, Thomas S. Worst, Philipp Nuhn

**Affiliations:** 1 Department of Urology and Urologic Surgery, University Medical Centre Mannheim (UMM), Medical Faculty Mannheim of Heidelberg University, Theodor-Kutzer-Ufer 1-3, 68167 Mannheim, Baden-Württemberg Germany; 2 Department of Medical Statistics and Biomathematics, Medical Faculty Mannheim of Heidelberg University, Mannheim, Germany

**Keywords:** Metastatic hormone-sensitive prostate cancer (mHSPC), Metastatic castration-resistant prostate cancer (mCRPC), Docetaxel, Cabazitaxel, Biomarkers, Prognosis, Treatment outcome

## Abstract

**Objectives:**

To assess the predictive and prognostic value of changes in longitudinal neutrophile-to-lymphocyte (NLR) ratios in men receiving taxane-based chemotherapy for metastatic prostate cancer (PC).

**Methods:**

Retrospective, unicentric cohort study of patients treated with either docetaxel for metastatic hormone-sensitive PC (mHSPC) or docetaxel or cabazitaxel for metastatic castration-refractory PC (mCRPC) at a tertiary referral hospital between 2010 and 2019. NLR ratios were calculated for each cycle. Next, slopes over the first three (NLR3) and over six cycles (NLR6) were calculated and analysed for biochemical/radiologic response and survival.

**Results:**

A total of 36 mHSPC (docetaxel), 118 mCRPC (docetaxel) and 38 mCRPC (cabazitaxel) patients were included. NLR3 was significantly associated with 1-year-survival, radiographic and biochemical response in mCRPC (docetaxel) in uni- and multivariable analyses. In mCRPC (docetaxel), positive NLR3s were associated with favourable 1-year-survival.

**Conclusion:**

This study demonstrated NLR3 as a prognostic marker in men receiving docetaxel for mCRPC. NLR3 might be a clinical tool to reflect the individual’s response to taxane-based chemotherapy. Thereby, NLR3 could complement existing biomarkers and help to early identify treatment failure before complications arise. Further prospective and multicentric studies are needed to extend and confirm the presented results.

## Introduction

Prostate cancer (PC) is the second most common solid cancer among the male population in the world [1]. Despite the recent growth in treatment options in metastatic stages, taxane-based treatment regimens remain an integral part of therapy algorithm in hormone-sensitive (mHSPC) and castration refractory prostate cancer (mCRPC) [2]. Due to its spectrum of side effects, the EAU guidelines recommend taxane-based chemotherapy for fit patients only [2]. To help tackle the more and more challenging task in clinical decision-making to choose the best treatment or sequence for every individual patient, several biomarkers have been developed and tested in metastatic PC. Inflammation fosters multiple cancer hallmarks [3]. Since a relation between inflammatory response and tumour progression has been shown [4], a lot of the studied biomarkers are inflammatory markers [5].

Prognostic biomarkers can roughly be divided into clinical and non-clinical markers. Clinical biomarkers for mHSPC and mCRPC include a variety of inflammatory and non-inflammatory routine laboratory markers, non-clinical markers include alterations in genomic sequencing of tumour tissue, circulating tumour cells or cell-free DNA [6–8]. Despite the abundance of available biomarkers, initial prostate specific antigen (PSA) and anaemia reflect the only laboratory biomarkers for overall survival (OS) next to presence of visceral metastases, pain, and bone scan progression in metastatic PC with docetaxel treatment [2]. For patients treated with cabazitaxel, the existing guidelines do not address prognostic biomarkers. Furthermore, the current guidelines list no biomarker as a predictor for failure of systemic treatment.

The number of prognostic biomarkers has led to the development of combined biomarkers. One of the most studied combined inflammatory markers in metastatic PC is the neutrophil-to-lymphocyte ratio (NLR) [9, 10]. This is also due to its convenience and ease of calculation [9]. Almost all of these studies in patients with mHSPC [8] or mCRPC [6, 11, 12] under docetaxel treatment or in patients with mCRCP under cabazitaxel treatment [13, 14] use NLR before treatment. We could only identify one study that examined a NLR kinetic over 3 cycles in patients with mCRPC under cabazitaxel [15].

It remains unknown if the NLR kinetic under therapy and thereby an individuals’ inflammatory response to taxane-based therapy is related to survival and therapy response and could serve as a novel biomarker for decision making.

In this study, we aimed to assess the NLR kinetics regarding their potential as prognostic and predictive biomarkers in patients with mHSPC and mCRPC under docetaxel therapy as well as in patients with mCRPC under cabazitaxel therapy.

## Methods

### Study population and data collection

Patients that were treated for either mHSPC or mCRPC between March 2010 and September 2019 at a certified prostate cancer centre of a tertiary university care centre (University Medical Centre Mannheim, Heidelberg University, Germany) were screened retrospectively. The Reporting Recommendations for Tumor Marker Prognostic Studies (REMARK) criteria were followed throughout this study. The inclusion criteria were (1) treatment with either docetaxel or cabazitaxel, (2) continuous treatment at the centre and (3) continuous androgen-deprivation therapy. Patients were excluded if taxane-based treatment was used as rechallenge. Previous androgen deprivation therapy was not an exclusion criterion Demographic and clinical information were extracted from the medical records in the centre and laboratory routine markers were recorded for all cycles received at the centre. The NLR ratio was calculated as follows:

$$\frac{{\mathrm{neutrophil}}\, {\text{count}}\left({\mathrm{reference}}\, {\text{range}}{:}\, {4{-}10\times 10}^{9}/\mathrm{L}\right)}{{\mathrm{lymphocyte}}\,{\text{count }}(\mathrm{reference}\, {\text{range}}{:}\, {1.1{-}3.2 \times 10}^{9}/\mathrm{L})}$$

For assessing the biochemical response, two endpoints were defined as response: PSA reduction by 30% and 50% at the end of the treatment compared to the PSA value at initial administration. Radiographic response to docetaxel was assessed by comparing baseline staging and available imaging 4–6 weeks after the last taxane-based treatment application. Response categories were complete response (CR), partial response (PR), stable disease (SD), or progressive disease (PD). CR, PR, and SD were analysed individually and grouped to classify treatment response. Patients gave informed consent for scientific use of their data (University of Heidelberg’s Ethics Committee II, Medical Faculty Mannheim, reference number 2015-549N-MA). Patient data was obtained from medical records and the local tumour register. Data cut-off was made in April 2020.

### Statistical analysis

**Fig. 1:**
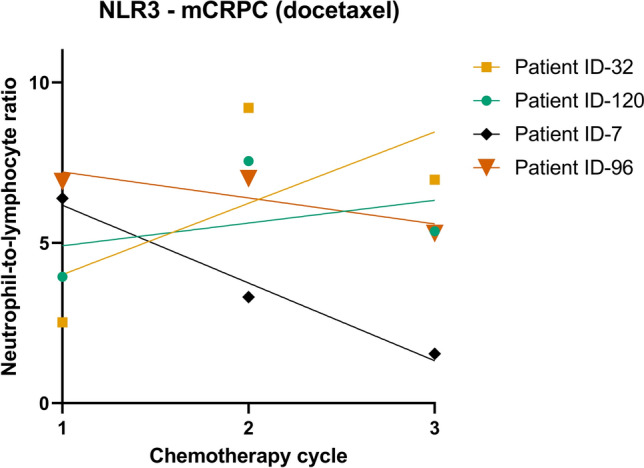
NLR values of 4 patients of the mCRPC (docetaxel) cohort over their first 3 cycles and their respective NLR slope (NLR3). *NLR3* NLR slope over the first 3 cycles, *mCRPC* metastatic castration-refractory prostate cancer, *ID* identification number

To characterize the cohort, descriptive characteristics were analysed as follows: Frequencies and proportions were calculated for categorical variables. For continuous variables, medians, and interquartile ranges (IQR) were calculated. After calculating the NLR, the NLR slopes were calculated for the first 3 (NLR3) or 6 therapy cycles (NLR6), where available. If the NLR could not be calculated for cycle 1 due to missing data, no slopes were calculated for the respective patient. As an example, the single NLR values and resulting NLR3 slopes are shown on 4 patients from the mCRPC cohort (docetaxel) in Fig. 1.

To assess the NLR slope as predictor for biochemical and radiographic response logistic regression analysis was performed. Additionally, patients were divided into groups with positive or negative NLR3 and NLR6 and evaluated for biochemical and radiographic response using X^2^ or Fisher’s exact test. Subsequently, significant variables were further analysed in univariable and multivariable logistic regression (backward selection), including age, visceral disease status, Gleason score, alkaline phosphatase (AP), haemoglobin (Hb), and PSA, to evaluate them as independent prognostic markers. For survival analysis uni- and multivariable Cox regression was conducted. In Cox regression analysis, C-indices were calculated according to Harrell et al. [16]. To evaluate the clinical-net benefit, decision-curve analyses were performed [17]. All tests comparing two groups were two-sided. The statistical significance level was set at α = 0.05 for the cohort containing n = 118 patients. Due to the small number of patients, the significance level for multiple models was set to alpha = 0.10. Calculations were performed using SAS^®^ software (SAS Institute Inc., Cary, North Carolina, USA, version 9.4). For Kaplan–Meier analyses GraphPad Prism9 (GraphPad Software, Inc, San Diego, CA, USA) was used.

## Results

**Fig. 2:**
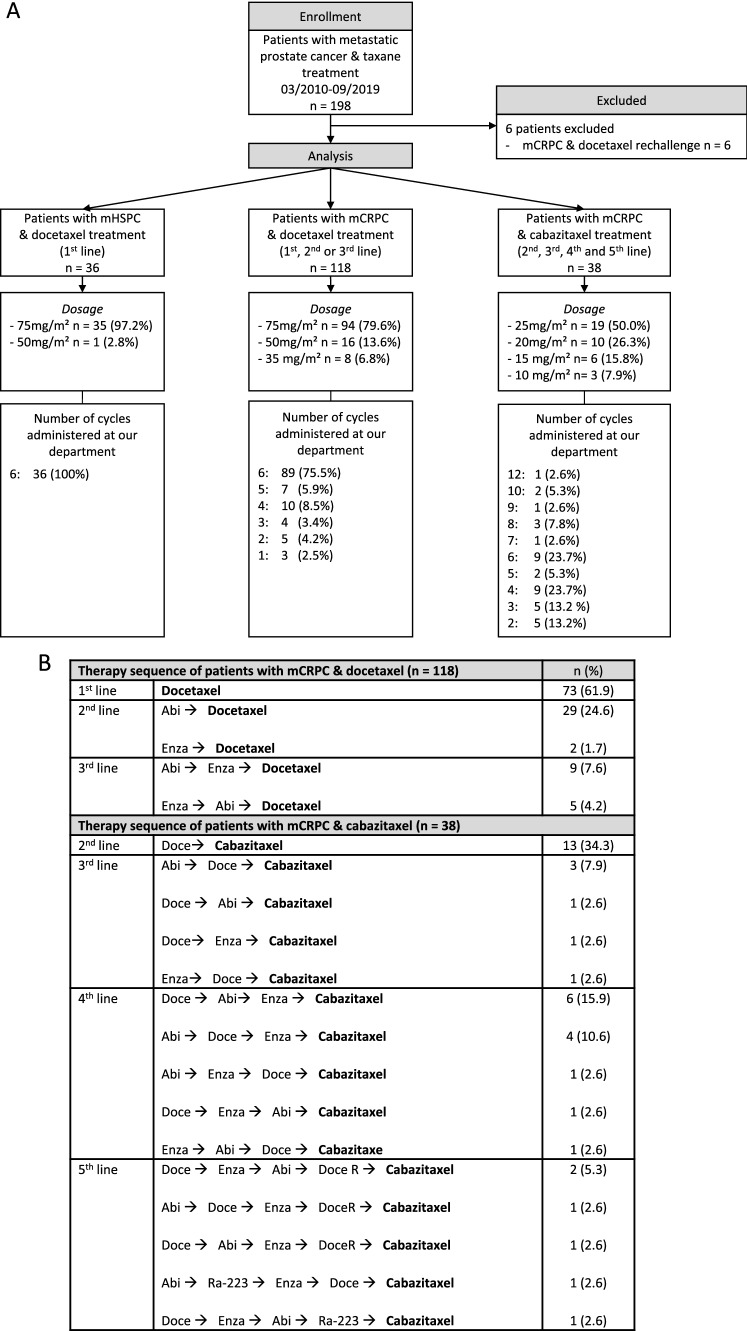
Flow-diagram and treatment information of the study cohorts. *mCRPC* metastatic castration-refractory prostate cancer, *mHSPC* metastatic hormone-sensitive prostate cancer, *Doce* docetaxel, *DoceR* docetaxel rechallenge, *Abi* abiraterone acetate, *Enza* enzalutamide, *Ra-223* Radium-223-dichloride

After exclusion for rechallenge, 192 patients were included in 3 cohorts. The mHSPC cohort treated with docetaxel in first line included 36 patients. One hundred and eighteen patients received docetaxel in mCRPC in 1st, 2nd or 3rd line and a total of 38 patients received cabazitaxel either in 2nd, 3rd, 4th, or 5th line. A detailed characterization of the cohorts—including number of cycles, dosage, and therapy sequence until docetaxel or cabazitaxel treatment—is shown in Fig. 2 and Table 1. In all three cohorts, most patients showed a positive NLR3 and NLR 6.

**Table 1:** Baseline characteristics of study cohorts

Characteristic	Cohort		
	mHSPC (docetaxel) (n = 36)	mCRPC (docetaxel) (n = 118)	mCRPC (cabazitaxel) (n = 38)
Age [years], median [IQR]	64 [60–70]	72 [65–76]	71 [63.8–74.3]
Gleason score ≥ 8 (n, %)^e^	27 (77.1)	67 (69.8)^e^	22 (62.9)^b^
PSA [ng/ml] at 1st cycle, median [IQR]	145 [27–304]	82.0 [23.4–266.5]^f^	92.5 [33.0–220.0]^k^
Lymphatic metastases (n, %)	22 (61.1)	68 (57.6)	23 (60.5)
Osseous metastases (n, %)	28 (77.8)	101 (85.6)	35 (92.1)
Visceral metastases (n, %)	6 (16.7)	29 (24.6)	7 (18.4)
Hb [g/dl], median [IQR]	13.8 [12.2–14.7]^a^	12.2 [10.2–13.3]^b^	11 [9.1–12.1]
AP [U/l], median [IQR]	122 [77–254]^b^	111.5 [74.8–230.0]^h^	150 [89.8–303.0]
Baseline NLR, median [IQR]	3.3 [2.3–4.3]^a^	3.9 [2.74–5.82]^g^	4.7 [3.5–7.4]^c^
NLR slope 3 cycles (NLR3), median [IQR]	2.27 [0.23–4.10]^c^	1.37 [-0.07–2.96]^i^	0.50 [-029–1.26]^c^
NLR slope 6 cycles (NLR 6), median [IQR]	0.14 [-0.51–1.14]^d^	0.33 [-0.08–0.92]^j^	0.23 [0.06–0.56]^l^
NLR3 positive (n, %)	19 (70.3)^c^	51 (71.9)^i^	19 (70.3)^c^
NLR6 positive (n, %)	10 (83.3)^d^	32 (69.6)^j^	10 (83.3)^l^
Follow-up [days] for OS, median [IQR]	889 [503–1337]	570 [316–1120]	270 [131–479]

*IQR* interquartile range, *OS* overall survival

^a^Data of 1 patient missing

^b^Data of 3 patients missing

^c^Data of 11 patients missing

^d^Data of 19 patients missing

^e^Data of 22 patient missing

^f^Data of 9 patients missing

^g^Data of 21 patients missing

^h^Data of 12 patients missing

^i^Data of 48 patients missing

^j^Data of 72 patients missing

^k^Data of 18 patients missing

^l^Data of 26 patients missing

In mHSPC, regression analyses of NLR3 and NLR6 did not yield significant results. Results are shown in Table 2A.

**Table 2:** Logistic regression and X^2^ or Fisher’s exact tests of NLR slopes over 3 (NLR3) and 6 (NLR6) cycles in the different cohorts **A** mHSPC cohort treated with docetaxel **B** mCRPC cohort treated with docetaxel **C** mCRPC cohort treated with cabazitaxel

A	mHSPC (docetaxel) (n = 36)					
	NLR3			NLR 6		
	NLR slope (regression analysis)		NLR slope (+ vs. −) (X^2^ or Fisher’s)	NLR slope (regression analysis)		NLR slope (+ vs. −) (X^2^ or Fisher’s)
	*p*	c	*p*	*p*	c	*p*
Survival (Cox regression)						
1-year survival	0.74	0.88	1.00^c^	n.a.^a^	n.a.^a^	n.a.^a^
3-year survival	0.99	0.36	1.00^c^	0.91	0.52	1.00^c^
5-year survival	0.72	0.43	0.57^c^	0.91	0.52	1.00^c^
Overall survival	0.72	0.43	0.57^c^	0.91	0.52	1.00^c^
Radiographic response (logistic regression)						
PR/CR	0.37	0.48	0.37^c^	0.87	0.54	1.00^c^
Biochemical response (logistic regression)						
PSA reduction ≥ 30%	n/a^b^		n/a^b^	n/a^b^		n/a^b^
PSA reduction ≥ 50%	n/a^b^		n/a^b^	n/a^b^		n/a^b^

B	mCRPC (docetaxel) (n = 118)					
	NLR3			NLR 6		
	NLR slope		NLR slope (+ vs. −)	NLR slope		NLR slope (+ vs. −)
	*p*	c	*p*	*p*	c	*p*
Survival (Cox regression)						
1-year survival	**0.05**	0.67	**0.03**^c^	0.88	0.53	0.43^c^
3-year survival	0.51	0.52	0.98^c^	0.49	0.57	0.25^c^
5-year survival	0.97	0.49	0.76^c^	0.45	0.67	0.07^c^
Overall survival	0.79	0.51	1.00^c^	0.57	0.64	0.15^c^
Radiographic response (logistic regression)						
PR/CR	**0.01**	0.69	0.15^c^	0.51	0.61	1.00^c^
Biochemical response (logistic regression)						
PSA reduction > 30%	0.06	0.63	0.21	0.48	0.53	0.74^c^
PSA reduction > 50%	**0.02**	0.67	0.26	0.31	0.55	0.32

C	mCRPC (cabazitaxel) (n = 38)					
	NLR3			NLR 6		
	NLR slope		NLR slope (+ vs. −)	NLR slope		NLR slope (+ vs. −)
	*p*	c	*p*	*p*	c	*p*
Survival (Cox regression)^d^						
1-year survival	0.28	0.57	1.00^c^	0.64	0.57	1.00^c^
3-year survival^d^	0.06	0.70	1.00^c^	0.42	0.59	0.52^c^
5-year survival^d^	0.06	0.70	1.00^c^	0.42	0.59	0.52^c^
Overall survival^d^	0.06	0.70	1.00^c^	0.42	0.59	0.52^c^
Radiographic response (logistic regression)						
PR/CR	0.11	0.73	1.00^c^	0.37	0.91	1.00^c^
Biochemical response (logistic regression)						
PSA reduction > 30%	0.43	0.43	0.66^c^	0.11	0.86	0.47^c^
PSA reduction > 50%	0.39	0.75	1.00^c^	0.13	0.85	0.46^c^

Bolded *p*-values denote statistical significance

*p* *p*-value, *c* C-Index, *PR* partial response, *CR* complete response, *NLR* neutrophile-to-lymphocyte ratio, *n/a* not applicable

^a^All patients with NLR slopes for 6 cycles showed survival > 1 year

^b^All patients in the mHSPC cohort receiving Docetaxel showed PSA reduction of ≥ 50%

^c^Fisher’s Exact Test

^d^Data for 3-year, 5-year and overall survival equal

**Fig. 3:**
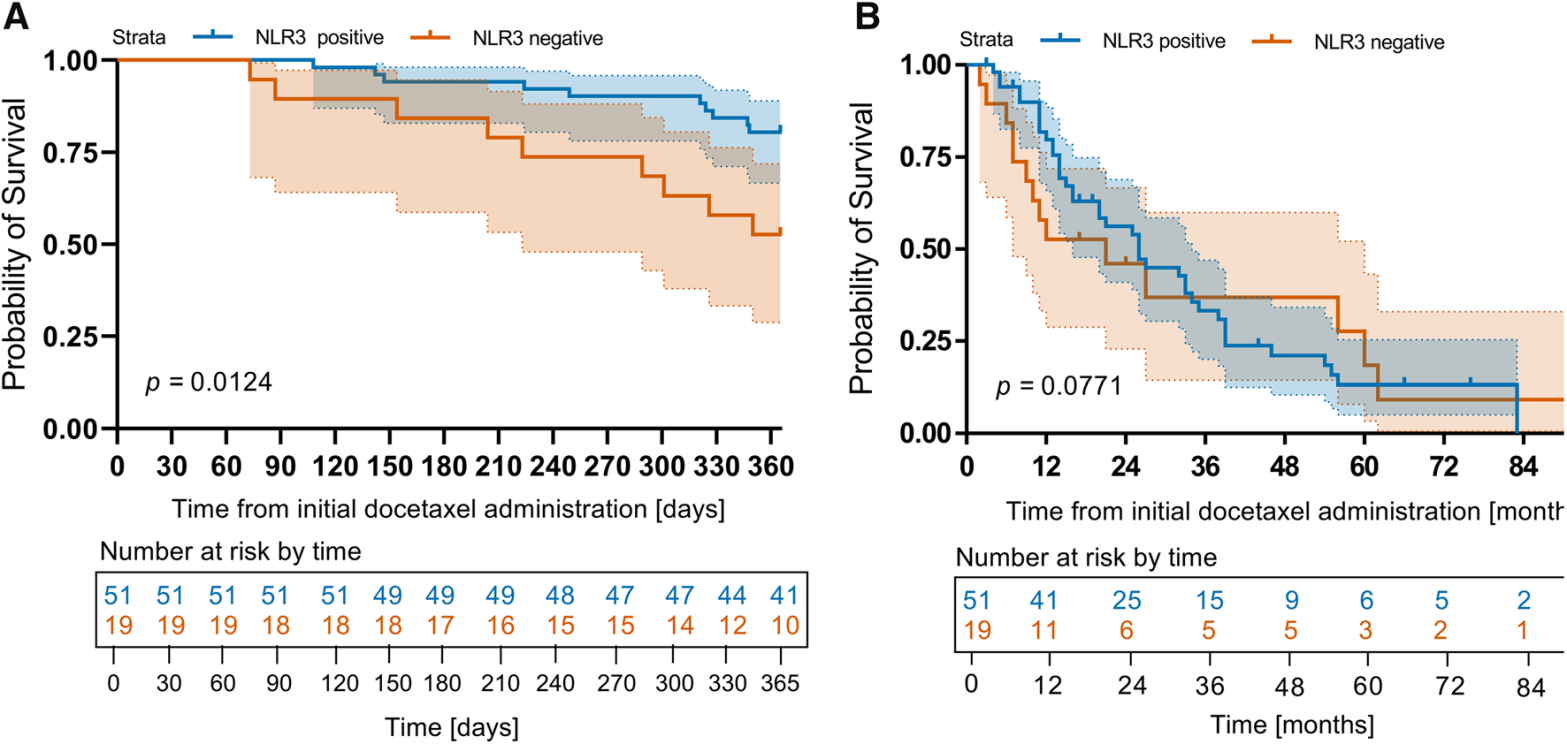
Kaplan–Meier analysis of **A** 1-year survival and **B** overall survival depending on positive or negative NLR slope over 3 (NLR3) cycles. *NLR* neutrophile-to-lymphocyte ratio, *NLR3* NLR slope over the first 3 cycles

In mCRPC treated with docetaxel, NLR3 was significantly associated with 1-year-survival in Cox regression analysis as well as radiographic response and PSA reduction of > 50%. When differentiating positive and negative NLR3, positive NLR3 was associated with 1-year-survival in Fisher’s Exact Test but did not show significant results for radiographic or biochemical results. Results are shown in Table 2B. Kaplan–Meier curves for 1-year survival and overall survival are shown in Fig. 3.

**Fig. 4:**
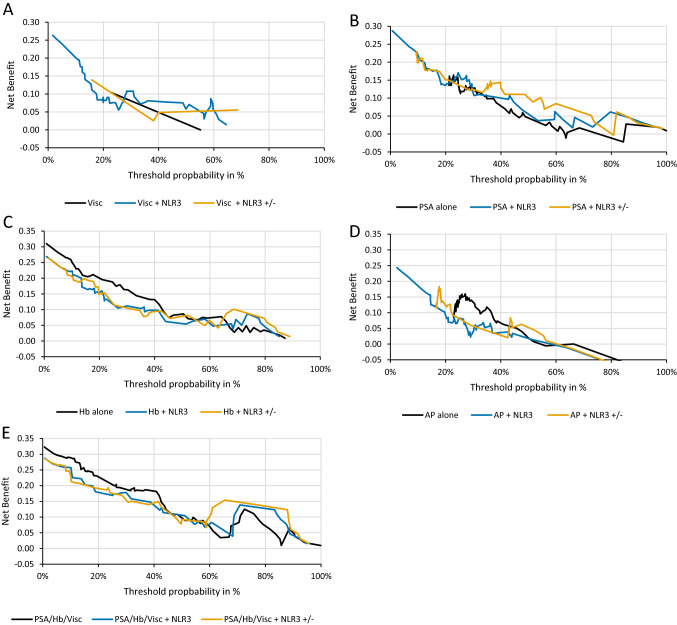
Decision curve analysis of multivariable models with the respective biomarker “NLR3” or “NLR3 positive or negative” **A** visceral metastasis, **B** PSA,** C** haemoglobin, **D** alkaline phosphatase and **E** PSA, haemoglobin, and visceral metastasis. *NLR3* NLR slope over the first 3 cycles, *NLR3 +/−* positive or negative slope over the first 3 cycles, *Visc* visceral metastasis, *Hb* haemoglobin, *AP* alkaline phosphatase

NLR3 remained significantly associated with 1-year survival, radiographic response, and PSA reduction > 50% in various multivariable regression models. Although NLR3 did not remain significant in the multivariable Cox regression model including all significant variables from univariable analysis, the C-index (AUC) in this model including NLR3 turned out highest. Results of uni- and multivariable regression analyses are shown in Table 3. In decision-curve analyses, the multivariable model consisting of PSA and NLR3 showed a clear net-benefit. In other multivariable models the net-benefit remained marginal. Results are shown in Fig. 4.

**Table 3:** Uni- and multivariable logistic and Cox-regression in the mCRPC cohort treated with docetaxel to detect variables associated with the **A** 1-year survival **B** radiographic response **C** and **D** biochemical response

A	1-year survival (Cox regression)							
	Univariable				Multivariable (n = 61)			
	HR	95% CI	*p*	c	HR	95% CI	*p*^a^	c
NLR3 in mCRPC (docetaxel) (n = 118)								
Age (per year)	1.02	0.97–1.06	0.45	0.52				
Visceral disease (yes vs. no)	3.25	1.69–6.29	**< 0.01**	0.63	2.30	0.76–6.96	0.14	0.83
Gleason Score ≥ 8 (yes vs. no)	2.23	0.85–5.88	0.10	0.58				
AP (per 100 units)	1.06	1.01–1.10	**0.01**	0.75	1.01	0.93–1.10	0.81	
Hb (per unit)	0.57	0.47–0.69	**< 0.01**	0.78	0.57	0.40–0.82	**< 0.01**	
PSA (per 100 units)	1.08	1.03–1.13	**< 0.01**	0.66	1.14	1.04–1.25	**< 0.01**	
NLR 3 neg. versus pos.	3.13	1.26–17.70	**0.01**	0.63	1.46	0.44–4.77	0.53	
NLR 3 (per unit)	0.80	0.65–1.00	**0.05**	0.65				

	HR	95% CI	*p*^a^	c	
Multivariable model 1 (n = 65)					
Visceral disease (yes vs. no)	2.96	1.14–7.71	**0.03**	0.76	
PSA (per 100 units)	1.10	1.06–1.26	**< 0.01**		
NLR 3 neg. versus pos.	2.67	1.03–6.92	**0.04**		
Multivariable model 2 (n = 69)					
NLR 3 (per unit)	0.81	0.65–1.02	**0.08**	0.66	
Visceral disease (yes vs. no)	2.95	1.18–7.41	**0.02**		
Multivariable model 3 (n = 65)					
NLR 3 (per unit)	0.82	0.67–1.01	**0.06**	0.71	
PSA (per 100 units)	1.14	1.05–1.24	**< 0.01**		
Multivariable model 4 (n = 65)					
NLR 3 (per unit)	0.79	0.60–1.04	**0.10**	0.65	
AP (per 100 units)	1.05	0.99–1.11	**0.06**		

B	Radiographic response (PR or CR; logistic regression)							
	Univariable				Multivariable (n = 59)			
	OR	95% CI	*p*	c	OR	95% CI	*p*^a^	c
Age (per year)	0.99	0.93–1.06	0.85	0.50				
Visceral disease (yes vs. no)	0.82	0.24–2.76	0.75	0.52				
Gleason Score ≥ 8 (yes vs. no)	0.99	0.31–3.19	0.98	0.50				
AP (per 100 units)	0.84	0.61–1.15	0.27	0.51				
Hb (per unit)	1.67	1.19–2.34	**< 0.01**	0.76	1.29	1.02–1.75	0.22	0.73
PSA (per 100 units)	0.93	0.78–1.11	0.41	0.50				
NLR 3 pos. versus neg.	5.25	0.62–44.38	0.12	0.61				
NLR 3 (per unit)	1.39	1.08–1.80	**0.01**	0.70	1.34		**0.04**	

C	PSA reduction 30% (logistic regression)							
	Univariable				Multivariable (n = 65)			
	OR	95% CI	*p*	c	OR	95% CI	*p*^a^	c
Age (per year)	0.98	0.93–1.03	0.45	0.55				
Visceral disease (yes vs. no)	0.51	0.20–1.27	0.14	0.56				
Gleason Score ≥ 8 (yes vs. no)	0.91	0.37–2.25	0.85	0.51				
AP (per 100 units)	1.00	0.91–1.09	0.91	0.53				
Hb (per unit)	1.44	1.15–1.80	**< 0.01**	0.70	1.42	1.06–1.92	**0.02**	0.73
PSA (per 100 units)	0.89	0.78–1.01	0.08	0.53				
NLR 3 pos. versus neg.	1.98	0.68–5.76	0.21	0.57				
NLR 3 (per unit)	1.28	1.01–1.64	**0.04**	0.65	1.10	0.92–1.55	0.19	

D	PSA reduction 50% (logistic regression)							
	Univariable				Multivariable (n = 69)			
	OR	95% CI	*p*	c	OR	95% CI	*p*^a^	c
Age (per year)	0.98	0.93–1.03	0.50	0.54				
Visceral disease (yes vs. no)	0.44	0.17–1.14	0.09	0.57				
Gleason Score ≥ 8 (yes vs. no)	0.78	0.32–1.91	0.58	0.53				
AP (per 100 units)	1.00	0.92–1.10	0.93	0.47				
Hb (per unit)	1.27	1.03–1.57	**0.02**	0.64	1.28	1.00–1.67	**0.09**	0.71
PSA (per 100 units)	0.91	0.80–1.04	0.16	0.55				
NLR 3 pos. versus neg.	1.86	0.63–5.50	0.26	0.56				
NLR 3 (per unit)	1.35	1.01–1.72	**0.02**	0.68	1.29	0.96–1.70	**0.05**	

Bolded *p*-values denote statistical significance

*HR* hazard ratio (for death), *OR* odds ratio (for meeting the respective endpoint in B, C & D), *p* *p*-value, *c* C-Index, *NLR* neutrophile-to-lymphocyte ratio, *NLR3* NLR slope over the first 3 cycles, *AP* alkaline phosphatase, *Hb* haemoglobin, *PR* partial response, *CR* complete response

^a^Due to the small number of patients, the significance level for multiple models was set to alpha = 0.10

For the mCRPC cohort treated with cabazitaxel, no significant results were reached, but Cox regression analysis of NLR3 revealed a *p*-value of 0.056 for 3-year, 5-year, and overall survival. Results are shown in Table 2C.

## Discussion

With advances in PC therapy more treatment options have become available. To determine the best timing for treatment change remains a difficult task especially in mCRPC, in which disease progression does not necessarily include PSA progression [18] and treatment discontinuation for a sole PSA-progression before 12 weeks of treatment is not recommended [19]. Therefore, additional intra-therapeutic biomarkers are necessary to identify treatment failure at an early stage and switch to a different therapy line before clinical complications occur. Intra-therapeutic changes in NLR could reflect treatment response. Whereas previous studies mainly measured delta-NLRs in two different time points (one pre-treatment and one during treatment), we calculated slopes from patients with 3 and/or 6 available NLR values. Therefore, we believe, that the NLR slopes might provide a more accurate reflection of the patients’ therapy response compared to a pre-treatment, post-treatment or two-timepoint delta-NLR.

We found NLR slopes over the first 3 cycles (NLR3) to be associated with 1-year survival, radiographic and biochemical response in mCRPC patients treated with docetaxel. In this cohort we could also show a slight increase of the C-indices in different multivariable models including NLR3 and partly a net-benefit in decision-curve analysis. In the mCRPC (cabazitaxel) cohort, NLR3 narrowly missed significance (*p* = 0.056) in logistic regression analysis when associated with 3-year-, 5-year- and overall survival. This might be due to the rather small cohort size.

Additionally, we found an increasing baseline NLR when comparing the three cohorts: mCRPC (cabazitaxel) > mCRPC (docetaxel) > mHSPC (docetaxel). This probably reflects the more advanced disease status, which leads to a higher inflammatory status in general. However, regardless of the cohort and their respective baseline NLR, NLR3 and NLR6 were positive in most patients in all three cohorts (range: 70.3–83.3%). Additionally, a positive NLR3 was associated with better 1-year-survival status in χ^2^-test and Kaplan-Meier analysis in the mCRPC (docetaxel) cohort. Furthermore, the NLR3 slopes decrease over the cohorts as disease status progresses.

Taken together, these findings allow to generate the hypothesis that a rising NLR could be interpreted as the reflection of the body’s inflammatory response to taxane treatment and thereby be a surrogate marker for prediction of treatment response and prognosis. This dynamic biomarker should be distinguished from high or very high pre-treatment NLR values, which are known to be associated with worse prognosis and OS in PC and other urologic and non-urologic malignancies [9, 20]: Baseline NLR has been shown as an easily available biomarker for treatment response in mHSPC and mCRPC, also in two of the above-mentioned cohorts [8, 21]. However, to the best of our knowledge evidence on NLR dynamics is sparse: In PC only one study exists, which analysed mCRPC patients treated with cabazitaxel: In this population a NLR decline > 30% after three cycles was identified as a predictor of favourable outcome for OS [15]. In localized PC, Jang et al*.* found a high postoperative NLR to be associated with unfavourable progression-free survival (PFS) and OS but could not find an association between delta-NLR and OS [22]. This also indicates that absolute (pre- or post-treatment) NLR values should be evaluated differently from delta-NLRs or NLR slopes.

In breast cancer, consistently low NLR values pre-treatment and 24–36 months after initial treatment have been associated with better prognosis [23]. Similar associations have been found in hepatocellular carcinoma and gastric cancer [24, 25]. These results do not match the results of our study. However, the different time points, different methodology and partially surgical character of interventions must be considered. Interestingly, in colon cancer an increase from pre- to post-surgical delta-NLR could be linked to a favourable clinical outcome by Li et al. [26]. It is not excluded, that different NLR dynamics have diverse prognostic meanings in various malignancies [26].

In summary, NLR is an easily available and cheap routine biomarker and its changes during chemotherapy could be a promising and accurate biomarker for PFS, OS and especially recognition of treatment failure in mCRPC treated with docetaxel.

## Limitations

The mHSPC (docetaxel) and mCRPC (cabazitaxel) cohort consist of a rather small sample size. Therefore, their results may not be generalizable. Additionally, the high number of dropouts for NLR3 potentially lowers the power of the multivariable analyses. Furthermore, with progressing treatment lines, the cohorts became more inhomogeneous. This is due to the individual courses of the disease. Moreover, no concomitant diseases were recorded. At the same time these limitations could also strengthen our findings and make them more robust and pragmatic. The fact that imaging was not evaluated according to Response Evaluation Criteria in Solid Tumours (RECIST) represents another limitation. Lastly, also to mention is the rather marginal net-benefit in decision-curve analysis of different multivariable models as well as the fact that the increase of c-indices in different multivariable models was small.

## Conclusion

This study shows the feasibility of NLR slopes and indicates their possible additional prognostic benefit, especially in mCRPC patients treated with docetaxel. In this cohort, NLR3 was significantly associated with radiographic and biochemical response as well as 1-year-survival. Whereas mHSPC is an early disease stage, in which almost all patients benefit from chemotherapy, in mCRPC NLR3 might reflect the individual’s response to taxane-based chemotherapy. Thereby, NLR3 could complement existing biomarkers and help to identify treatment failure early before complications arise. Further prospective and multicentric studies are needed to extend and confirm the presented results.

## Data Availability

The datasets generated during and/or analysed during the current study are available from the corresponding author on reasonable request.
